# Wound Healing Improvement with PHD-2 Silenced Fibroblasts in Diabetic Mice

**DOI:** 10.1371/journal.pone.0084548

**Published:** 2013-12-20

**Authors:** Xiongliang Zhang, Xiaoyu Yan, Liang Cheng, Jiezhi Dai, Chunyang Wang, Pei Han, Yimin Chai

**Affiliations:** Department of Orthopedics, Sixth People's Hospital, Shanghai Jiao Tong University, Shanghai, China; National Institutes of Health, United States of America

## Abstract

**Background:**

Hypoxia-inducible factor 1α is the central regulator of the hypoxia-induced response which results in the up-regulation of angiogenic factors. Its activity is under precise regulation of prolyl-hydroxylase domain 2. We hypothesized that PHD2 silenced fibroblasts would increase the expression of angiogenic factors, which might contribute to the improvement of the diabetic wound healing.

**Materials and Methods:**

50 dB/db mice were employed and randomly assigned into five groups with 10 mice in each: group 1 (untreated cell), group 2 (PHD2 silenced cell), group 3 (L-mimosine treated cells), group 4 (nontargeting siRNA treated cells) and group 5 (sham control). Fibroblasts were cultivated from the dermis of mice in each group and treated with PHD2 targeting siRNA, L-mimosine and non-targeting siRNA respectively. A fraction of the fibroblasts were employed to verify the silencing rate of PHD2 after 48 hours. The autologous fibroblasts (treated and untreated) labeled with adenovirus-GFP were implanted around the wound (Φ6mm), which was created on the dorsum of each mouse. The status of wounds was recorded periodically. Ten days postoperatively, 3 mice from each group were sacrificed and wound tissues were harvested. Molecular biological examinations were performed to evaluate the expressions of cytokines. 28 days postoperatively, the remaining mice were sacrificed. Histological examinations were performed to evaluate the densities of GFP+ cells and capillaries.

**Results:**

The expression of PHD2 reduced to 12.5%, and the expressions of HIF-1α and VEGFa increased significantly after PHD2 siRNA treatment. With the increasing expressions of HIF-1α and VEGFa, the time to wound closure in group 2 was less than 2 weeks. Increased numbers of GFP+ cells and capillaries were observed in group 2.

**Conclusion:**

PHD2 siRNA treatment not only increased the expression of HIF1α and VEGFa, but also improved the fibroblast proliferation. These effects might contribute to the improvement of the diabetic wound healing.

## Introduction

 In patients with diabetes, wounds tend to heal much more slowly than those in normal individuals, even well-controlled diabetics are at an increased risk of post-surgical wound complications[1]. Delays in wound healing often result in infection, chronic ulceration, and possible amputation of extremities. Of all amputations in diabetic patients, 85% are preceded by a foot ulcer which subsequently deteriorates to a severe infection or gangrene[2]. So diabetic-induced wound healing impairment is a significant challenge to surgeons. It not only increases patient experienced pain and extends hospital stay, but also places a significant burden on the health care system. Impaired wound healing is a major complication of the 23 million people in the USA with diabetes, and financial and medical burdens demand new treatments for wound healing[3].

 Hypoxia-inducible factor 1α (HIF-1α) is the central regulator of the hypoxia-induced response. In hypoxia, HIF-1α translocates to the nucleus and promotes the transcription of a variety of effectors such as VEGF and FGF-2, promoting angiogenesis and vasculogenesis[4,5]. The diabetic wound is hypoxic, yet HIF-1α levels have been shown to be reduced in diabetic wound healing[6]. So manipulation of responses to hypoxia is desirable in diabetic wound healing. Increasing or stabilizing the expression of HIF-1α has proven sufficient to induce angiogenesis, even in normoxia[7]. But directly elevating the HIF1-α expression would result in some adverse effects[8]. Moreover, HIF1-α activity is controlled by HIF-prolyl hydroxylase domain (PHD). The increased HIF1-α may also augment the expression of PHDs accordingly[9] and reduce the total effect. Furthermore, gene-modified or viral-transfected overexpression of chemokines would introduce novel genetic materials into the living body and increase the risk of potential viral infection. Therefore, these methods were experimental rather than clinical because of the associated ethical issues. 

 Prolyl hydroxylase domain (PHD) proteins play a critical role in oxygen homeostasis[10]. Oxygen-dependent PHDs negatively regulate HIFs and, crucially, confer its oxygen sensitivity. In the presence of oxygen, PHD2 hydroxylates HIF-1α on two specific proline residues, which results in its destruction. In hypoxia, PHD2 is missing its cosubstrate (oxygen), rendering it inactive, then HIF-1α becomes stabilized, and results in the up-regulation of angiogenic factors such as vascular endothelial growth factor (VEGF), fibroblast growth factor (FGF)-2, and angiopoeitin-2 thereby promoting neovascularization. HIF-prolyl hydroxylase-2 (PHD2) serves as a crucial oxygen sensor and may therefore play an important role in response to hypoxia[11]. So the PHDs are consequently an attractive therapeutic target[12,13].

 Studies have shown that small interfering RNAs (siRNAs) can be used to suppress the transcription of specific gene sequences in cells, thereby inhibiting the production of a specific protein product[14,15]. Fibroblasts are proved to be able to improve the wound healing in diabetic animals[16,17]. We hypothesized that PHD2 silenced fibroblasts implantation in diabetic wound would improve wound healing and the progress would be associated with improved neovascularization. 

## Materials and Methods

### 1. Ethics.

 All experimental animal procedures were approved by the Institute of Animal Care and Use Committee of School of medicine, Shanghai Jiaotong University (Permit Number: SYXK 2011-0128) and performed in accordance with the Regulations of Laboratory Animal Care. All surgery was performed under sodium pentobarbital anesthesia, and all efforts were made to minimize suffering.

### 2: Animals and group division

 Autosomal recessive mutation derived diabetes in db/db mouse strain is spontaneously diabetic mice which was identified as a mutation in the leptin receptor gene[18]. Total 50 Lepr db/db mice of 6 weeks (Model Animal Research Center of Nanjing University, Nanjing, China) with 35-40 gr each were employed and randomly assigned to 5 groups with 10 mice in each. Group 1 (implanted with untreated cell), group 2(implanted with PHD2 silenced cell), group 3(implanted with L-mimosine treated cells), group 4(implanted with nontargeting siRNA treated cells), group 5(sham control). Group 3 served as the positive control and group 4 served as the negative control.

### 3: Murine fibroblast cultivation and identification

 Each mouse along with sample of fibroblasts isolated was carefully labeled accordingly for the next autotransplantation step. Primary murine dermal fibroblasts were isolated according to standard procedures[19]. In brief, 10×10 mm^2^ abdominal skin of db/db mice was removed and incubated with 0.1% trypsin and 0.02% EDTA in PBS overnight at 4°C. Then, the dermis, mechanically separated from the epidermis, was minced and incubated with 400 U/ml of collagenase I (Invitrogen, USA) for 1 h at 37°C. Tissue debris was removed by centrifugation, and fibroblasts were cultured in RPMI 1640 Medium (Invitrogen, USA) containing 50 μg/ml Na-ascorbate (Sigma-Aldrich, USA), 2 mM glutamine and antibiotics (Gibco, Invitrogen, USA) and 10% fetal calf serum (Hyclone, Thermo Scientific, Australia) at 37 °C in 5% CO2. 

 Fibroblasts were used in passage 2–4 and labeled with replication-defective adenovirus-GFP (Courtesy from Dr. Ling Du, Shanghai Key Laboratory of Orthopedic Implants, Shanghai Ninth People's Hospital) with 150 multiples of infection (MOI = 150) before implantation. The incisions were sutured with 4-0 mersilk (Johnson & Johnson, USA). After revival from anesthesia, the animals were returned to animal center and kept as usual.

 To identify the population of fibroblasts cultivated, the cells were trypsinized, washed twice with phosphate buffered saline (PBS), fixed by 4% formaldehyde, then perforated by Triton X-100, and immunostained for 30 mins on ice with Alexa Fluor 488 conjugated monoclonal antibody against vimentin (Cell Signaling Technology, Danvers, Massachusetts, USA). Flow cytometric analyses were performed by utilizing Beckmann coulter Navios fluorescence-activated cell sorter.

### 4: PHD2 gene silencing

 A commercial siRNA kit was applied to silence the PHD2 gene in murine fibroblasts. According to the manufacturer’s protocol, siGENOME SMART pool (Dharmacon, Thermo, USA) was added into the substrate to silence the PHD2 gene in fibroblasts. L-minosine is an inhibitor of PHDs and was employed as the positive control (at 1 mmol/L concentration). siGENOME Non-Targeting siRNA Pool #1 (Dharmacon, Thermo, USA) was used as negative control. DharmaFECT reagent 1 (Dharmacon, Thermo, USA) was used as the transfection reagent at the following concentration: 0.2 μL DharmaFECT/100 μL transfection medium. The incubation lasted for 24h, then normal medium was applied to continue the cultivation. 48h later, Real-Time Quantitative Polymerase Chain Reaction was performed to examine the relative expression of PHD2, HIF-1α and VEGFa and verify the silencing rate of RNA interference. β-actin was used as the internal control. 

### 5: Cell viability assay

 A Cell Counting kit-8 (Dojindo,Japan) was used to examine cell viability according to the manufacturer's instructions[20]. Fibroblasts were seeded at 2x10^3^ cells/well in 96-well culture plates and then treated with PHD2 siRNA, non-targeting siRNA and L-mimosine respectively at 37°C for 24 hours in a humidified incubator containing 95% air and 5% CO2. Blank control (containing only RPMI 1640 medium) and normal control (containing cells and 1640 medium but without reagents) were established. After 12, 24, 48, 72, 96 hours, the 10 μl CCK-8 solution was added to each well and incubated for 2 h in an incubator. The absorbance was measured by using a Synergy HT multidetection microplate reader (Bio-Tek, Winooski, VT, USA) at a wavelength of 450 nm. 

### 6: Wound model and fibroblast implantation

 An established murine model of excisional wound healing was employed in the study with minor modification[21]. Briefly, wounds (Φ6mm) were created on the dorsum of the mice and the perimeter of the wounds was marked with India ink. Then the wounds were fixed to deep tissue with 4-0 mersilk (Johnson & Johnson, USA) to prevent the wound from contraction and allow the wounds to heal by secondary intention. 4×10^5^ treated or untreated autologous fibroblasts were injected subcutaneously around the wound after the surgery immediately and the implantations were repeated once at day 5. Group 5 did not receive cell implantation and served as the sham control group. The wounds were then covered with a transparent sterile occlusive dressing (3M, USA). The status of the wounds was observed periodically and recorded by a digital camera (T200, Sony, Japan) at a same distance and same focus. Wound areas were calculated in square millimeter by using ImageJ Software (version 1.46; National Institute of Health, USA). The results were expressed as percentage of the healed area relative to the original wound area of the animals and calculated with the following formula: wound closure=(original area-wound area)/original area*100%). At day 10 postoperatively, 3 mice from each group were sacrificed and the wound tissues were harvested for molecular biological examinations. The remaining mice were kept until 28 days postoperatively and sacrificed for histological examinations.

### 7: mRNA expression for PHD2, HIF-1α and VEGFa in tissue

 A full thickness skin specimen of 0.5*1 cm in size from the wound was harvested (n=3 for each group) at day 10 postoperatively and homogenized immediately at 4°C. Total RNA from skin samples was isolated by using Trizol reagent (Invitrogen, Life technology, USA), according to the manufacturer’s protocol. 1μg RNA sample was reverse-transcribed with oligo dt_15_ by AMV (Promega, USA). Real-time PCR (7500 Sequence Detection System, ABI, Invitrogen, USA) amplifications were performed by using the Power SYBR Green PCR Master Mix reagent (Invitrogen, Life technology, USA). Primer pairs used were listed in Table 1. β-actin mRNA was used as an internal control. Quantification is expressed as fold induction to untreated cells implantation group.

**Table 1:** Primers used in Real-Time Quantitative Polymerase Chain Reaction.

Gene Name	Genbank_ID	Primer Sequence	Product length
β-actin	NM_007393.3	Forward: ACGTTGACATCCGTAAAGACC Reverse: AGCCAGAGCAGTAATCTCC	107bp
PHD2	NM_053207.2	Forward: CCACTGGCACTCAACTAACTCA Reverse:CCGAGTTCATTTAGTGCCCGTCA	114bp
HIF-1α	NM_010431.2	Forward: AAGCCCTCCAAGTATGAGCAC Reverse: GCCACTGTATGCTGATGCCTTA	214bp
VEGFa	NM_001025250.3	Forward: GAACCAGACCTCTCACCGGAA Reverse: GACCCAAAGTGCTCCTCGAAG	136bp

### 8: Western Blot Analysis

 Equal amounts (10–30 μg) of protein extracts from wound tissue of the animals (n=3 for each group) at day 10 postoperatively were loaded and separated by SDS- PAGE using 12% acrylamide gradients. The membranes were incubated with monoclonal antibodies against mouse PHD2 (Epitomcis, UK), HIF-1α (Abcam, USA), VEGF (Abcam, USA). Signals were detected with horseradish peroxidase-conjugated goat anti-mouse or goat anti-rabbit immunoglobulin G. Proteins were transferred to nitrocellulose membranes which were then incubated in the primary antibody solution (anti-DNP 1:150) for 2 hrs, followed by incubation with the secondary antibody solution (1:300) for 1 hr at room temperature. The washing procedure was repeated five times within 30 mins. Immunoreactive bands were visualized by enhanced chemiluminescence (Amersham Biosciences) which was then exposed to Biomax L film (Kodak). For quantification, enhanced chemiluminescence signals were digitized using Labwork software (UVP).

### 9: Micro-vessel density evaluation

 The specimens were fixed in 10% buffered formalin, embedded in paraffin, cut into 5 μm-thick sections, and stained with anti-CD31 (Abcam, Cambridge, MA, USA) and colored by DAB staining. The vascular density was confirmed by calculating the CD31+ capillaries in 20 random fields at 400×magnification according to the previously described method[22]. Normal mouse liver tissue was used as positive control. Negative controls consisted of samples in which the primary antibodies were replaced with PBS. The counting was performed by two experienced reviewers blinded to the research.

### 10: Fluorescence microscope examination

 The frozen sections of the specimen were examined with a fluorescence microscope (Eclipse 80i, Nikon, Japan). GFP+ cells were searched for and counted in 20 random fields at 200×magnification. The counting was performed by two experienced reviewers blinded to the research.

### 11: Statistical analysis

 All experiments were performed in triplicate and repeated three times. Quantitative data are expressed as means ± SD. Statistical analysis was adequately performed by analysis of variance followed by Tukey multiple comparison procedure. Statistical analysis was performed using SPSS for Windows ver. 13.0 (SPSS, inc., Chicago, IL, USA). A probability value of p≤0.05 was considered statistically significant.

## Results

### 1: Fibroblasts cultivation, identification and labeling

**Figure 1:**
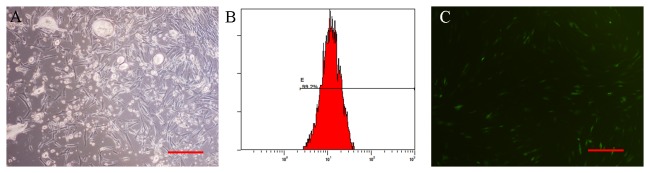
Murine fibroblasts cultivation, identificaion and labeling. A: Fibroblasts developed a characteristic bipolar spindle-shape at 7 days after seeding (100×magnification). B: Flow cytometric analyses showed that vimentin was expressed in most of the cells. C: Characteristic green fluorescence was observed in fibroblasts labeled with adenovirus-GFP under the fluorescence microscopy (100×magnification). The scale bars in right lower corner represent 200 μm.

 Primary fibroblasts were isolated from the dermis of the mice and developed a characteristic bipolar spindle-shape at day 7 (Figure 1A) . Flow cytometric analyses showed that vimentin was expressed in most of the cells (Figure 1B). Characteristic green fluorescence was observed in the fibroblasts incubated with adenovirus-GFP when cells were examined by fluorescence microscopy (Figure 1C).

### 2: mRNA expressions after treatment in vitro

**Figure 2:**
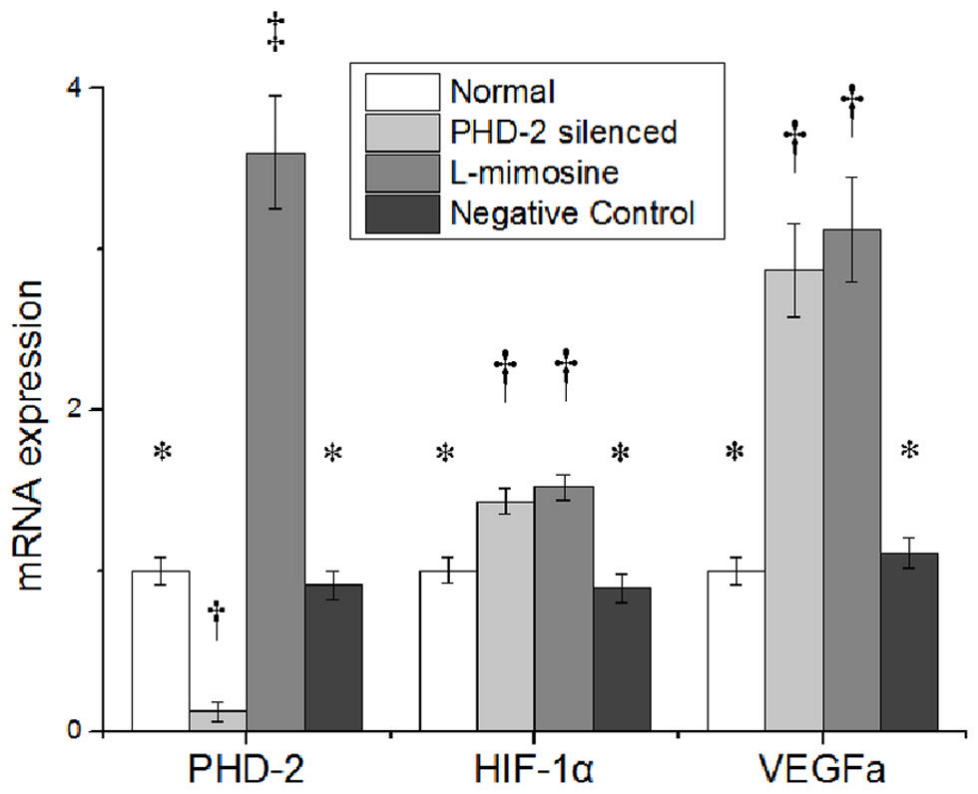
Prolyl hydroxylase domain 2 (PHD2), Hypoxia inducible factor 1 (HIF-1α), Vascular Endothelial Growth Factor (VEGFa) gene expression at 48h after treatment in vitro. siRNA, small interfering RNA. Symbols (*,†,‡) indicate significant difference (at .05 level) by Tukey multiple comparison procedure. * vs. †, * vs. ‡ , † vs. ‡, p≤0.05. There is no significant difference between groups labeled with same symbol.

 Forty eight hours after treatment, mRNA expression of PHD2 was sharply reduced to 12.5% in group 2 (PHD2 siRNA treated cells), indicating the efficacy of RNA interference (p≤0.05). However, the mRNA expression of PHD2 in groups 3 (L-mimosine treated cells) increased by over threefold(p≤0.05). But there was no significant difference between group 1 and group 4(p>0.05) . (Figure 2 left part)

 The expression of HIF-1α increased significantly in group 2 and group 3 (p≤0.05), but there was no difference between group 1 and group 4 or between group 2 and group 3 (p>0.05) (Figure 2 middle part).

 The expression of VEGFa dramatically increased in group 2 and group 3 by nearly threefold (p≤0.05), but there was no significant difference between group 1 and group 4 or between group 2 and group 3 (p>0.05). (Figure 2 right part)

### 3: Cell viability assay

**Figure 3:**
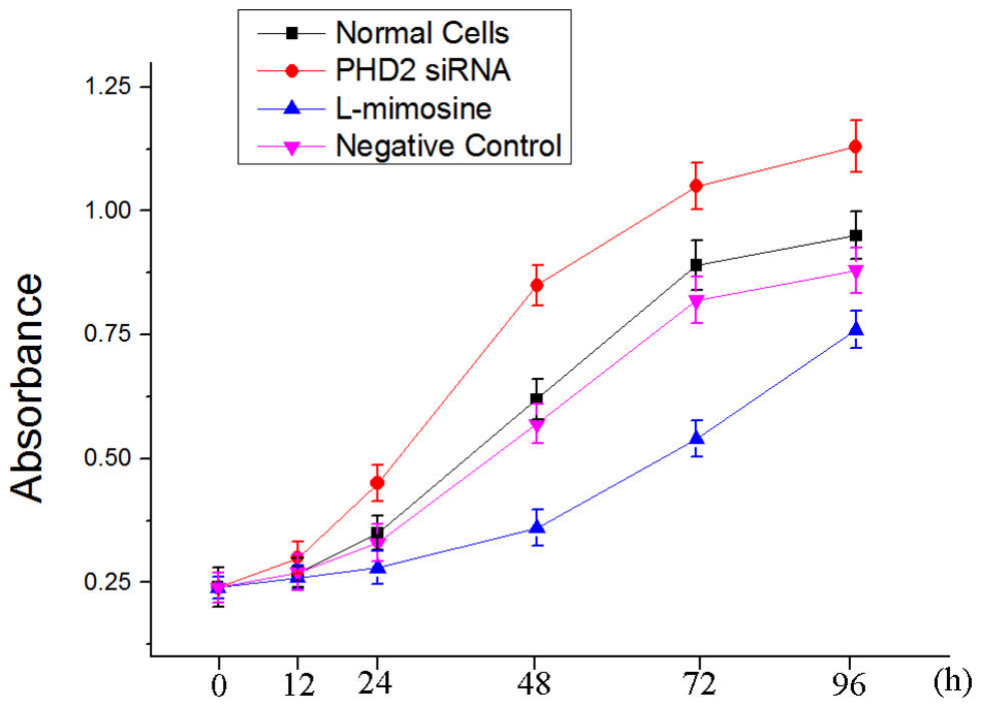
Cell viability assay for fibroblast proliferation: The proliferative ability of PHD2 silenced fibroblasts was superior to that of the cells in other groups. The proliferative ability of fibroblasts treated by L-mimosine was inferior in the four groups.

 After 4 days cultivation, the viability of the fibroblasts was superior in group 2(P≤0.05). The proliferation ability of the cells in group 1 was similar to that in group 4 (p>0.05). However, the viability in group 3 was inferior to those of other three groups (p≤0.05) . (Figure 3)

### 4: wound healing

**Figure 4:**
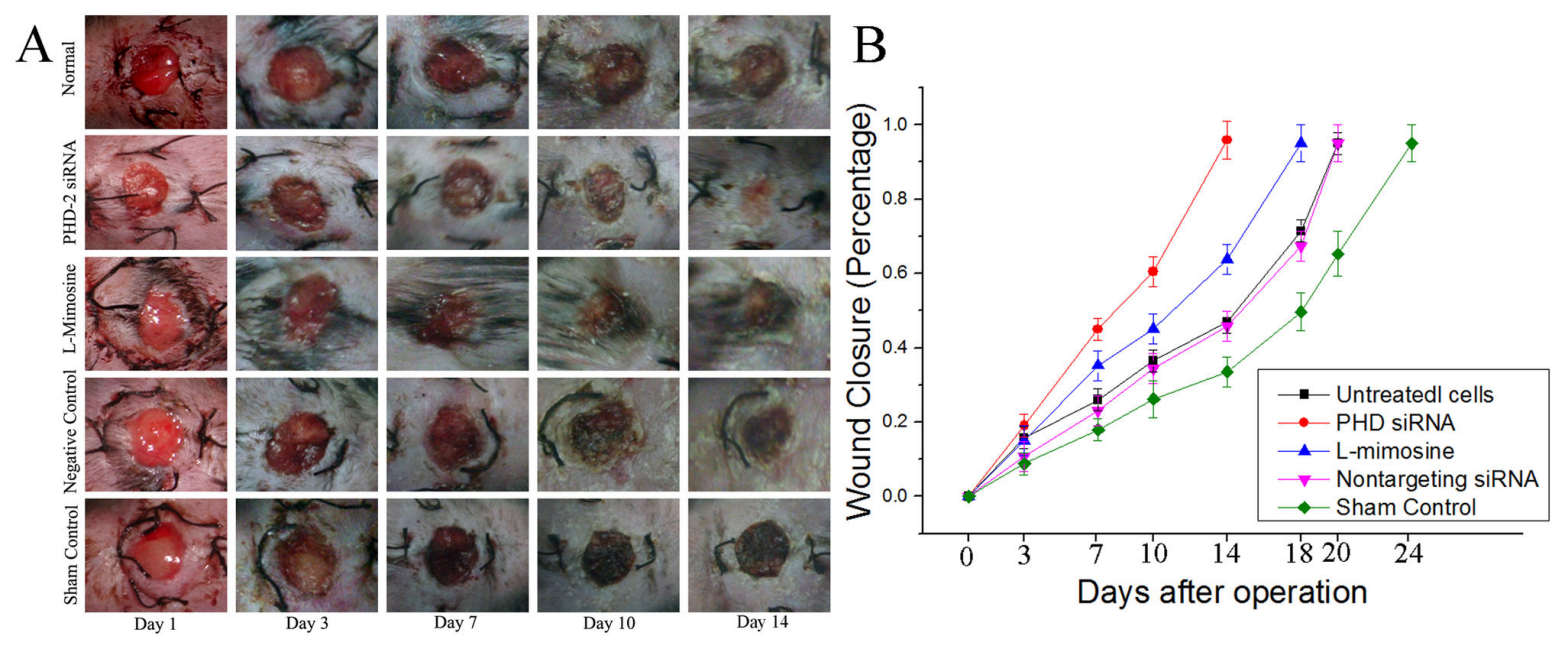
Wound healing of the diabetic wound. A: Gross imaging of the wound healing. The wounds in group 2 healed within 2 weeks. The wound healing in group 3, which was implanted with fibroblasts treated by L-mimosine, was also accelerated when compared with the rest three groups. B: Wound closure quantified. The time to wound closure in group 1 (implanted with untreated fibroblasts ) was around 21 days. In group 2 (implanted with PHD2 siRNA treated fibroblasts), the time was shortened to less than 2 weeks with statistic significance (p≤0.05). The time in group 3 (implanted with L-mimosine treated cells) was around 18 days (P≤0.05). In group 1 and group 4, the wounds healed in 3 weeks. In group 5(sham control group), the wounds needed nearly 4 weeks to heal.

 Gross images of the wounds were taken at day 1, 3, 7, 10, 14 revealed an accelerated rate of time to wound closure in the Group 2 (treated by PHD2 silenced fibroblasts) (Figure 4A). This observation was confirmed by photometric analysis at day 0, 3, 7, 14, 18, 20, and 24 whereby PHD2 silenced fibroblasts treated diabetic wounds experienced a statistically significant increase in wound closure at every time point examined (Figure 4B). The wounds in group 2 healed within 14 days (>95%) . In contrast, the wounds in group 3 were only 63% healed at the same time point. However, the healing of wounds in group 3 was still superior to the rest three groups (p≤0.05), and the time to wounds closure in the groups with cell implantation (group 1-4) was shorter than that of sham control (group 5). Overall, the time to wound closure in group 2 was shortened by 4 days, 6 days and 10 days when compared with group 3, group 1 or 4, and group 5 respectively.

### 5: mRNA expression for PHD2, HIF-1α and VEGFa in vivo

**Figure 5:**
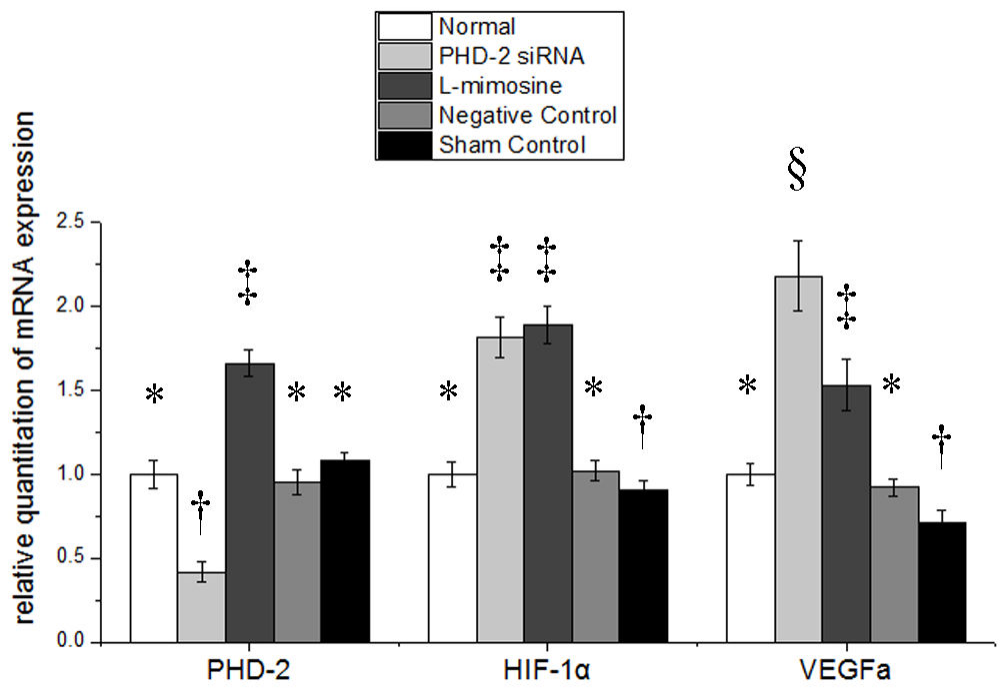
Prolyl hydroxylase domain 2 (PHD-2), Hypoxia inducible factor 1α (HIF-1α), vascular endothelial growth factor a(VEGFa) gene expression in vivo on days 10. siRNA, small interfering RNA. Symbols (*,†, ‡, §) indicated significant difference (at .05 level) by Tukey multiple comparison procedure. * vs. other groups, p≤0.05. There is no significant difference between groups labeled with same symbol.

 10 days postoperatively, the expression of PHD2 in group 2 was sharply reduced in group 2 and remarkably higher in group 3 than in other groups, but there was no significant difference among group 1, 4 and 5. (Figure 5)

 The expressions of HIF1α in group 2 and 3 were notably higher than the rest three groups, and slightly reduced in group 5, but there was no significant difference between group 1 and 4.

 The expression of VEGFa increased in group 2 and group 3. Moreover, the expression in group 2 was even higher than that in group 3. But it reduced in group 5. But there was no significant difference between group 1 and 4.

### 6: Western blot analysis

 The relative density of PHD2 reduced significantly in group 2 and group 4, and increased in group 5(P≤0.05). There was no significant difference between group 1 and 3(P>0.05).

 The relative density of HIF1 increased in group 2 and 3(P≤0.05), and slightly reduced in group 5, but there was no significant difference between group 1 and 4 or group 2 and 3(P>0.05).

**Figure 6:**
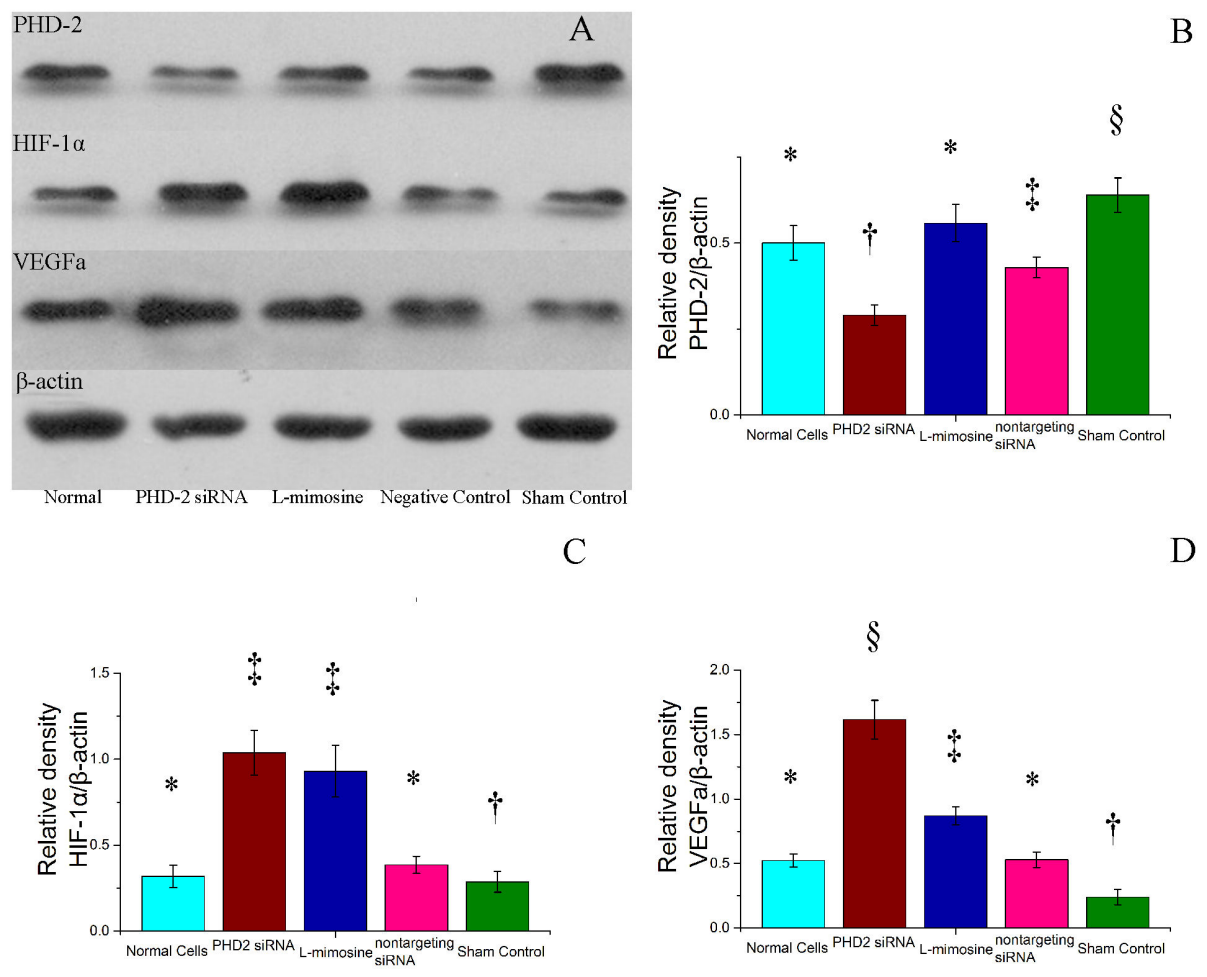
Assessment of the expression of Prolyl hydroxylase domain 2 (PHD-2), Hypoxia inducible factor 1α (HIF-1α), vascular endothelial growth factor a (VEGFa) in vivo. A: gross images of western blot results. B: Quantified PHD2 expression relative to β-actin. C: Quantified HIF-1α expression relative to β-actin. D: Quantified VEGFa expression relative to β-actin . siRNA, small interferingRNA. Symbols (*,†, ‡, §) indicated significant difference (at .05 level) by Tukey multiple comparison procedure. * vs. other groups, p≤0.05. There is no significant difference between groups labeled with same symbol.

 The expression of VEGFa increased in group 2 and group 3. It was highest in group 2 and lowest in group 5. There was no significant difference between group 1 and 4.(Figure 6)

### 7: Histological and micro-vessel density evaluation

**Figure 7:**
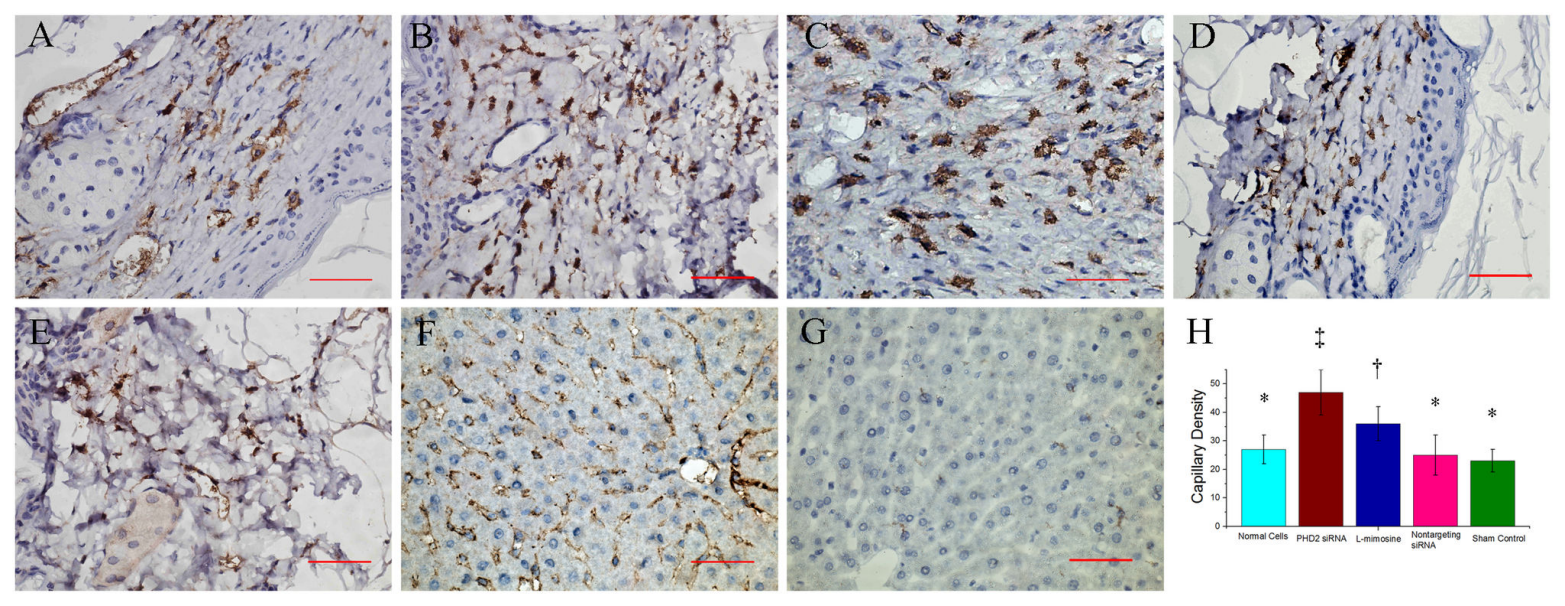
Representative images of immunohistochemistry stain by CD31 (400×magnification). A: Wound tissue in group 1 (implanted with untreated fibroblasts). B: Wound tissue in group 2 (implanted with fibroblasts treated by PHD2 siRNA), showing blood vessels stained by CD31 with higher density. C: Wound tissue in group 3 (implanted with L-mimisine treated fibroblasts). D: Wound tissue in group 4 (implanted with negative control fibroblasts). E: Wound tissue in group 5 (without cell implantation), showing low density of blood vessel with small diameter. F: CD31 immunohistochemistry stain for murine liver tissue, served as positive control. G: negative control of CD31 immunohistochemistry stain. H: Comparison of microvessel density among different groups. Symbols (*,†, ‡) indicated significant difference (at .05 level) by Tukey multiple comparison procedure. * vs. †, * vs. ‡ , † vs. ‡, p≤0.05. There is no significant difference between groups labeled with same symbol. The scale bars in right lower corner represent 50 μm.

 Immunohistochemistry stain targeting the cell surface marker CD31,an endothelial biomarker, revealed increased microvessel densities (MVD) in group 2 and group 3. The MVD in group 2 was even higher than in group 3. The MVD in group 5 was lowest among the five groups. There was no statistic significant difference among group 1, 4 and 5 (Figure 7).

**Figure 8:**
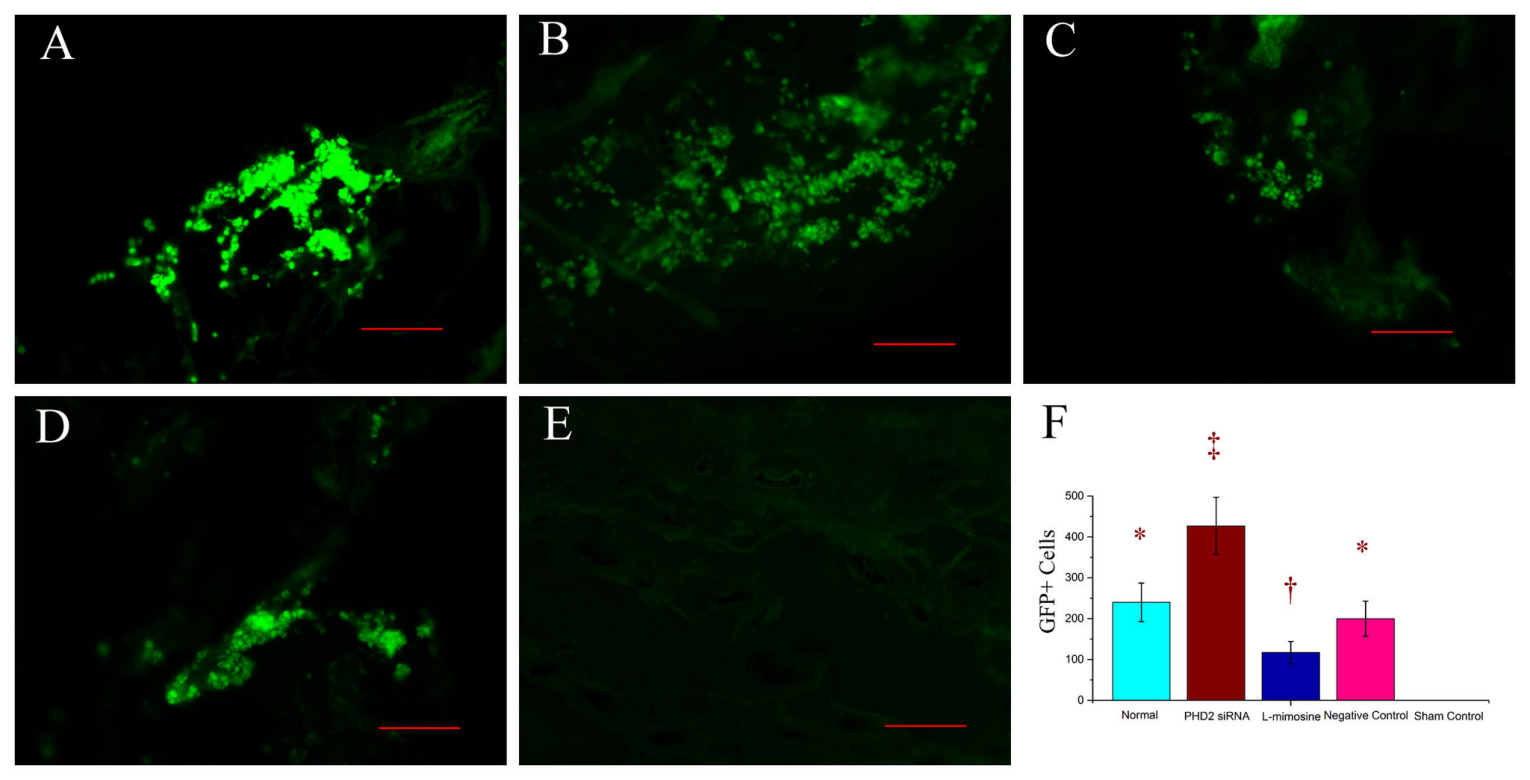
Representative images of fluorescence microscope examination (200×magnification), showing the implanted fibroblasts labeled with adenovirus-GFP. A: GFP+ cells in group 1. B: GFP+ cells in group 2, the number of the GFP positive cells was higher than the other groups. C: GFP+ cells in group 3. The number of GFP+ cells was the lowest in four groups. D: GFP+ cells in group 4. E: GFP+ cells in group 5, there was no GFP+ cells observed. F: Comparisons of GFP+ cells among different groups. Symbols (*,†, ‡) indicated significant difference (at .05 level) by Tukey multiple comparison procedure. * vs. †, * vs. ‡ , † vs. ‡, p≤0.05. There is no significant difference between groups labeled with same symbol. The scale bars in right lower corner represent 100 μm.

 Fluorescence microscope examination showed an increased number of GFP+ cells in group 2, and a reduced number of GFP+ cells in group 3, but there was no significant difference between group 1 and group 3. There was no GFP+ cell observed in group 5 (Figure 8).

## Discussion

 The aim of the study was to investigate the effect of the PHD2 silenced fibroblasts implantation on the healing of diabetic wound in an animal model. In the present study, we demonstrated that: 1) The fibroblasts implantation could improve the healing of diabetic wound. 2) PHD2 silenced fibroblasts implantation could further improve the healing of diabetic wound. 3) PHD2 targeting RNA interference could elevate the level of HIF1α and VEGFa, and even increase the proliferation of fibroblasts, which may contribute to the improvement of the wound healing in diabetic mice. So we believe that PHD2 silenced fibroblasts implantation is an effective way to improve the diabetic wound healing. 

 Wound healing requires the integration of complex cellular and molecular events in successive phases of inflammation, granulation tissue formation, and re-epithelialization. In particular, dermal fibroblasts play essential roles in the repair of skin wounds through remodeling of the wound bed by synthesis of new extracellular cell matrix and growth factors and the formation of thick actin bundles[23]. Impaired wound healing in diabetics is a well-documented phenomenon, and aberrant fibroblast function contributes to this process[6,24,25]. So how to improve the function of fibroblasts is important to the healing of the diabetic wound.

 Prolyl hydroxylase domain 2 (PHD2) has been implicated in several pathways of cell signaling, especially in the degradation of HIF-1α[26]. PHD2 is also crucial negative regulators of cell cycle progression and cell proliferation. Kalucha demonstrated that loss of PHD2 in keratinocytes stimulates wound closure by prompting skin epithelial cells to migrate and proliferate[27]. Another research showed that silencing the PHD2 gene by RNA interference significantly enhanced hypoxia-induced endothelial cell proliferation[28]. In the present study, we observed that the number of GFP+ fibroblast increased in the PHD2 siRNA treated group. Although the real mechanism of PHD2 in cell proliferation is not clear, but researches indicated that PHD2 may be associated to the survival of cells[29,30]. Another study demonstrated that the phenomenon was associated with the unique expression of β3 -integrin in a HIF1α-dependent manner[27]. But the proliferation of another cell type in mice was more likely associated to HIF2α rather than HIF1α[31], which implied that the proliferative effect of PHDs may be cell type specific.

 Study revealed that PHDs inhibitors could increase the production of vascular endothelial growth factor in dental pulp-derived cells[32]. Although the prolyl hydroxylase inhibitors may also suppress the activity of PHD-2 as the PHD-2 targeting RNA interference does, but the prolyl hydroxylase inhibitors seem to reduce the viability of cells[33]. The prolyl hydroxylase inhibitors may exert the inhibitory effect via different approaches[34]. Western blot results showed that although the HIF-1α level was similar in group 2 and group 3, the VEGFa level was higher in group 2 than that in group 3. This phenomenon could be partly explained by the nonspecific inhibitory effect of L-mimosine because of its extensive biological effects, yet further studies are needed to testify this speculation. The nonspecific inhibitory effect of the PHD2 inhibitors may be a crucial disadvantage for its application in wound healing. However, PHD2 targeting siRNA treatment is superior to PHD2 inhibitors because of its accurate targeting ability, which may overcome and even reverse the defect of PHD inhibitors and make the therapy more attractive. 

 RNAi reagents employed in the present study are composed of naked siRNA and tend to be degraded in human plasma in a few minutes due to the presence of RNases and fast renal clearance[35-37]. So they are not suitable for topical use in wound[38]. However, employing the autologous fibroblasts as the ideal vector to exert the effect of PHD2 siRNA may avoid the defect and bring extra advantage of the cell transplantation at the same time. Because of the depletion of siRNA, the effect of RNA interference could last only several days[39]. In our pilot study, this effect could only last utmost 5 days. In order to sustain the effect on improving wound healing, we repeated the cell implantation at day 5.

 Although research indicated that vector-based RNAi may induce a prolonged gene silencing effect compared with siRNA-mediated RNAi[40]. However, perhaps most importantly from a clinical perspective, siRNA-mediated gene silencing was temporary, as expected given the nature of siRNA, and that no trace of gene knockdown could be detected 3 weeks after the siRNA application. So it is safe to be applied in clinical. 

## Conclusion

 In summary, we concluded that PHD2 silenced fibroblasts could improve diabetic wound healing, likely through a vasculogenic and proliferative mechanism. The impairment of wound healing in diabetes can be attenuated by the up-regulation of pro-angiogenic pathways via RNAi. The pathway, stabilization of HIF-1α by silencing PHD2, is central to the improvement of diabetic wound healing through a neovascular mechanism. Interventions that promote angiogenesis may provide an important strategy to mitigate the aberrant injury response in diabetes. In the light of these findings, PHD2 silenced fibroblasts might offer a potential role in the healing of the diabetic wound.
